# Fostering inclusion in medical training: accommodations for hearing loss

**DOI:** 10.1093/occmed/kqae094

**Published:** 2024-11-27

**Authors:** M Belingheri, M A Riva, S Brambilla, M E Paladino

**Affiliations:** School of Medicine and Surgery, University of Milano-Bicocca, Monza 20900, Italy; Division of Occupational Health, Fondazione IRCCS San Gerardo dei Tintori, Monza 20900, Italy; School of Medicine and Surgery, University of Milano-Bicocca, Monza 20900, Italy; Division of Occupational Health, Fondazione IRCCS San Gerardo dei Tintori, Monza 20900, Italy; Agency for Health Protection of the Brianza, Monza 20900, Italy; School of Medicine and Surgery, University of Milano-Bicocca, Monza 20900, Italy; Division of Occupational Health, Fondazione IRCCS San Gerardo dei Tintori, Monza 20900, Italy

## Abstract

This article explores two case reports of healthcare students with hearing impairments in an Italian university and the accommodations provided during their clinical training. The first student, with bilateral sensorineural hearing loss, used the Contec Visual Electronic Stethoscope CMS-VE, which amplifies the sound up to 32 times and supports earphones while wearing hearing aids. The second student, with profound bilateral sensorineural hearing loss and cochlear implants, utilized the Littmann CORE Digital Stethoscope, which amplifies the sound up to 40 times and transmits it via Bluetooth. Both students successfully acquired essential clinical skills, demonstrating that tailored technological accommodations can significantly enhance learning outcomes. This article underscores the critical role of early disability detection and the implementation of support strategies by occupational health physicians. These case reports highlight the importance of innovative solutions in promoting inclusion and ensuring equal opportunities in medical education and future workplaces.

## Teaser

This article presents two case reports of healthcare students with hearing impairments who utilized two different advanced digital stethoscopes during their clinical training. Highlighting the importance of tailored technological accommodations, it demonstrates how these innovations enabled students to successfully acquire essential clinical skills. The findings underscore the role of early disability detection and support strategies in fostering inclusion and ensuring equal opportunities in medical education and the workplace once they have completed their studies.

**Key learning points**

**What is already known about this subject:**

- Students with hearing impairments face significant challenges in acquiring clinical skills, particularly in auscultation, due to the reliance on auditory abilities.
- Technological advancements have led to the development of digital and electronic stethoscopes designed to assist individuals with hearing impairments.
- There is a need for effective accommodations in medical training to ensure equal educational opportunities for students with disabilities.

**What this study adds:**

- Tailored technological accommodations enabled students to successfully acquire essential auscultation skills comparable to their peers without disabilities.
- The use of the two different digital stethoscopes significantly improved the clinical training outcomes for students with hearing impairments.

**What impact this may have on practice or policy:**

- Occupational health evaluations should prioritize early identification of sensory impairments to develop effective support plans.
- Policies promoting the integration of assistive technologies in medical education can ensure equal opportunities and better preparedness for students entering the healthcare workforce.

## Background

In recent years, the inclusion of students with disabilities in higher education has become an increasingly important issue [1]. Ensuring that all students, regardless of their physical or sensory impairments, have equal access to educational opportunities is a fundamental goal for educational institutions.

Pre-internship medical evaluations play a crucial role in identifying students’ needs for accommodations. These evaluations help to detect any physical or sensory impairments that might affect a student’s ability to perform essential clinical tasks. By identifying these needs early, educational institutions can implement appropriate accommodations, ensuring that all students have the support they need to succeed in their clinical training [2].

In the field of healthcare education, inclusion presents unique challenges. Clinical skills, such as auscultation, rely heavily on sensory perception, particularly auditory and tactile senses. For students with hearing impairments, mastering these skills using traditional equipment can be exceptionally difficult. This necessitates the development and implementation of innovative solutions to ensure that all students can achieve the necessary competencies to become effective healthcare professionals.

However, advancements in technology have led to the development of electronic and digital stethoscopes designed to assist individuals with hearing impairments [3].

These tools have been critical in reducing barriers and facilitating the inclusion of students with disabilities in medical training programs. For instance, universities in Italy have been actively enhancing their support services and technological aids to ensure accessibility and inclusivity in education [4].

This article explores two case reports of healthcare students with hearing impairments who utilized particular stethoscopes during their clinical training. These cases highlight the importance of accommodations in promoting inclusion and ensuring that all students can succeed in their medical education.

## Case presentations

During the fitness-to-work assessment in an Italian university before starting the clinical internship, two students reported hearing loss and the consequent use of hearing aids (first student) and cochlear implants (second student).

**Figure 1.**
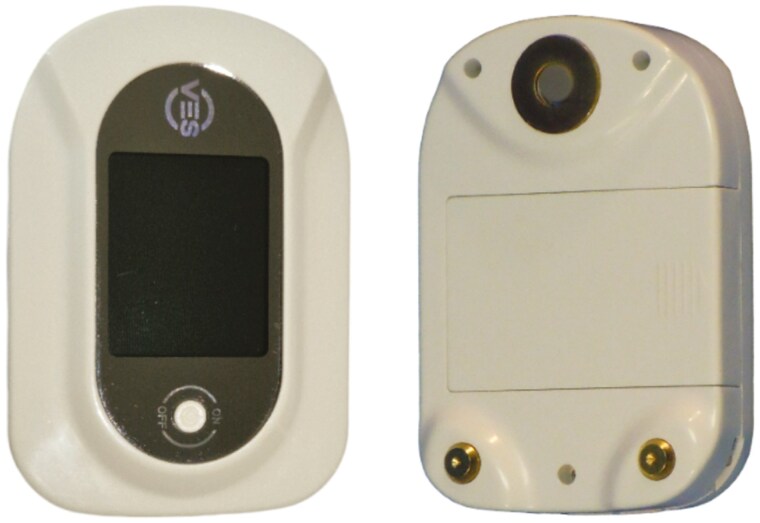
The Contec Visual Electronic Stethoscope—CMS-VE.

The first student was a 21-year-old healthcare student with bilateral sensorineural hearing loss—moderate in the left ear and severe to profound in the right ear. His hearing loss was progressive and had been present since childhood, necessitating the use of hearing aids for daily auditory assistance. The student had difficulty in using a traditional acoustic stethoscope, as he had to remove his hearing aids to auscultate, resulting in difficulty hearing sounds. This compromised his ability to measure blood pressure and listen to heart and lung sounds effectively, skills required by the internship and future profession. The solution proposed was the Contec Visual Electronic Stethoscope, CMS-VE (Figure 1). This digital stethoscope amplifies the sound up to a maximum of 32 times and volume settings include 16 steps of programmable sound levels. Users can listen to the digital audio signal through the built-in headphone jack. With this tool, the student was allowed to use earphones and adjust the volume. In addition, the student could connect circumaural headphones that completely wrap around and surround the ears, allowing the student to continue wearing their hearing aids.

A training session of approximately 1 hour was conducted with an occupational health physician. The training covered the operation of the stethoscope (basic functions, recharging and amplification adjustment) and included a practical session on specific clinical tasks, such as blood pressure measurement. Additionally, the student could contact the occupational health physician for further training if any issues arose with the use of the stethoscope during the internship.

After this brief period of training to the new tool, he successfully achieved all the required learning outcomes and skills. With the Contec Visual Electronic Stethoscope, CMS-VE, the student successfully performed all necessary auscultatory tasks, achieving results comparable to his peers without hearing impairments. Throughout his internship, he demonstrated excellent auscultation skills and passed all clinical evaluations.

The second student was a 20-year-old healthcare student with profound bilateral sensorineural hearing loss, who had received bilateral cochlear implants at the age of 3 years old. Using a conventional stethoscope was problematic as well, and it was not possible to use the same system employed for the first student due to the nature of the cochlear implants and the impossibility to use earphones. Cochlear implants bypass the normal acoustic hearing process to directly stimulate the auditory nerve, making it impossible for the student to use earphones, which were necessary for the stethoscope used by the first student.

**Figure 2.**
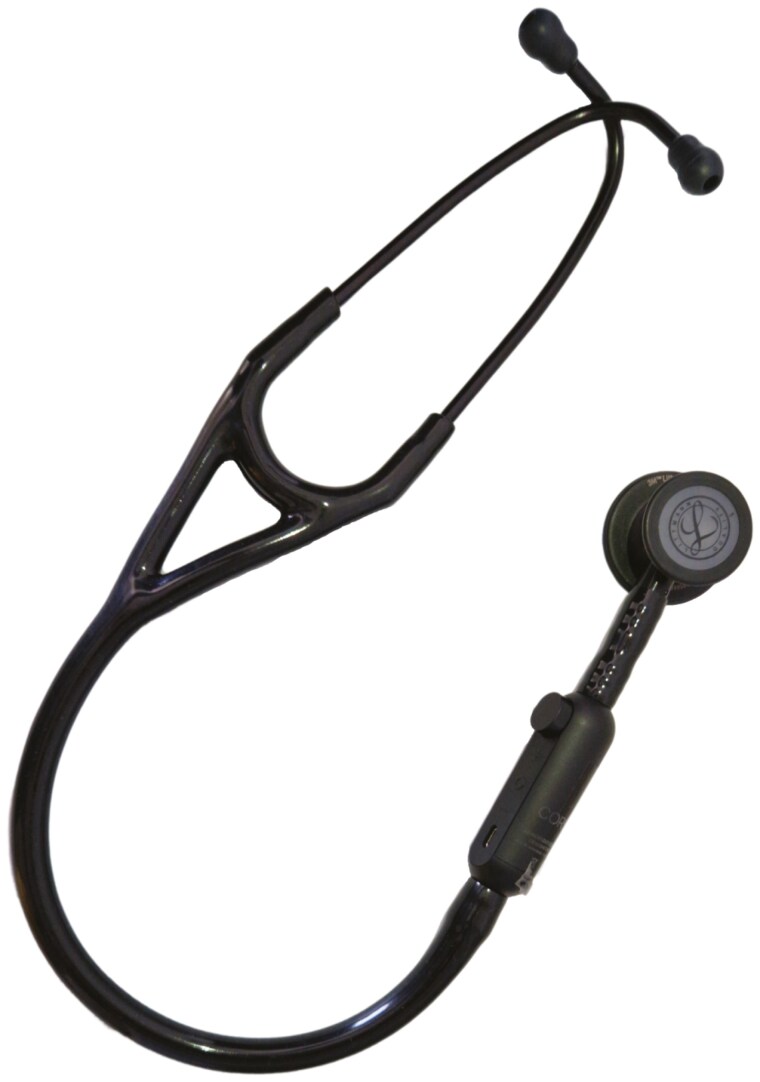
The Littmann CORE Digital Stethoscope.

Therefore, a Bluetooth stethoscope, the Littmann CORE Digital Stethoscope, was selected (Figure 2). This stethoscope amplifies the sound up to 40 times, has an active noise cancellation function and can transmit the auscultation sounds to the cochlear implants via a mobile app, ensuring clear and effective sound transmission. Even in this case, the student received a similar brief training session focussed on the new tool, particularly in blood pressure measurement. The student accurately performed auscultatory activities, such as distinguishing heart and respiratory sounds. For the second student as well, these skills were required by the internship and future profession. He successfully passed all clinical evaluations and continued her training without further issues.

## Discussion

Providing accommodations for students and employees with disabilities is vital for fostering inclusion and equal opportunities. In healthcare education, digital stethoscopes and other assistive technologies improve the learning experiences of students with hearing impairments, enabling them to achieve their full potential.

Electronic and digital stethoscopes are designed for healthcare professionals seeking enhanced auscultation, particularly in noisy environments. Some of these stethoscopes offer additional features beyond sound amplification, such as the ability to detect real-time electrocardiogram (ECG) waveforms and calculate heart rates. They also integrate with specific apps, allowing users to record, share and analyse sounds for more detailed assessments. Although not specifically created for individuals with hearing impairments, tailored electronic and digital stethoscopes can significantly enhance the clinical training and performance of these students. These accommodations ensure that all students, regardless of their disabilities, can acquire the essential skills required to become competent healthcare professionals.

These case reports demonstrate the significant role of technology in overcoming auditory barriers in healthcare education. Adapting stethoscopes to the needs of students with hearing impairments enables them to acquire essential competencies without discrimination. Providing such accommodations is a crucial step towards greater inclusion and equality in educational and professional settings. Furthermore, the same technologies can obviously be used during the work activities of these future healthcare professionals.

Moreover, the inclusion of students with disabilities in higher education, particularly in Italian universities, highlights the ongoing efforts to create a supportive and accessible learning environment. Initiatives such as the development of specialized technological aids, individualized support services and the establishment of technical committees on accessibility and inclusion are crucial in ensuring that students with disabilities receive the necessary support [4]. The use of assistive technologies such as the Visual Electronic Stethoscope and Bluetooth stethoscopes are examples of how innovative solutions can facilitate the inclusion of students with sensory impairments in demanding fields like healthcare. Recent studies comparing conventional and amplified stethoscopes have shown that amplified stethoscopes provide better amplification of both normal and abnormal heart sounds across various frequencies. This is particularly important for healthcare professionals with hearing impairments, as these stethoscopes can help mitigate the limitations of traditional acoustic stethoscopes and improve diagnostic accuracy [3].

While advanced stethoscopes offer significant advantages for students with hearing impairments, some challenges should be considered. First, the cost of these devices is higher than that of traditional stethoscopes—though generally not prohibitive—and for some students or institutions with limited budgets, this may be a concern. Additionally, the size and portability, particularly with circumaural headphones, can be cumbersome in some clinical settings, and the reliance on battery power raises concerns about battery life and the need for regular recharging. However, these challenges were not significant enough to hinder the effective use of the stethoscopes in our cases.

The role of occupational health physicians is critical in ensuring that students undergoing clinical internships are thoroughly evaluated for any disabilities that may affect their training. Pre-internship medical evaluations are essential for identifying students’ needs for accommodations early on. These evaluations help detect physical or sensory impairments that might hinder a student’s ability to perform essential clinical tasks, thus allowing educational institutions to implement appropriate accommodations.

The early identification and management of disabilities through medical evaluations enable the creation of tailored support plans that ensure all students have the necessary resources to succeed in their clinical training. This proactive approach not only helps in mitigating potential challenges but also fosters a more inclusive and supportive learning environment.

Occupational health physicians play a vital role in this process by conducting comprehensive assessments and collaborating with educational institutions to develop and implement effective accommodation strategies. Indeed, in the cases presented, although both students had hearing loss, they could not use the same digital stethoscope model. Individual assessment of disability characteristics was necessary to identify the best tool for each student.

The expertise of occupational health physicians is essential to ensure that students with disabilities receive the support they need, thus promoting equal opportunities and enhancing the overall quality of healthcare education. Additionally, proper management of disabilities during university will assist students in handling their disabilities in the workplace once they have completed their studies and become healthcare professionals.

## References

[1] Toutain C. Barriers to accommodations for students with disabilities in higher education: a literature review. J Postsecond Educ Disabil 2019;32:297–310.

[2] Riva MA , Bava M, D’Orso MI, De Vito G, Cesana G. How can medical students suffering from hearing loss auscultate their patients? Med Lav 2014;105:315–316. 25078999

[3] Alanazi A , Atcherson S, Franklin C, Bryan M. Frequency responses of conventional and amplified stethoscopes for measuring heart sounds. Saudi J Med Med Sci 2020;8:112. 32587492 10.4103/sjmms.sjmms_118_19 PMC7305673

[4] ANVUR Agenzia Nazionale di Valutazione del sistema Universitario e della Ricerca. Gli studenti con disabilità e DSA nelle università italiane [Internet]. 2022. https://www.anvur.it/wp-content/uploads/2022/06/ANVUR-Rapporto-disabilita_WEB.pdf (June 2024, Date accessed).

